# *trans*-Diaqua­bis­[2,5-bis­(pyridin-2-yl)-1,3,4-thia­diazole]nickel(II) bis­(tetra­fluoridoborate)

**DOI:** 10.1107/S1600536811026420

**Published:** 2011-07-09

**Authors:** Fouad Bentiss, Frédéric Capet, Michel Lagrenée, Mohamed Saadi, Lahcen El Ammari

**Affiliations:** a Laboratoire de Chimie de Coordination et d’Analytique (LCCA), Faculté des Sciences, Université Chouaib Doukkali, BP 20, M-24000 El Jadida, Morocco; b Unité de Catalyse et de Chimie du Solide (UCCS), CNRS UMR 8181, ENSCL, BP 90108, F-59652 Villeneuve d’Ascq Cedex, France, Université Lille Nord de France, F-59000 Lille, France; c Laboratoire de Chimie du Solide Appliquée, Faculté des Sciences, Université Mohammed V-Agdal, Avenue Ibn Battouta, BP 1014, Rabat, Morocco

## Abstract

The bidentate 1,3,4-thia­diazole ligand, namely, 2,5-bis­(2-pyrid­yl)-1,3,4-thia­diazole (denoted *L*), untested as a polydentate ligand, has been found to form the monomeric title complex, [Ni(C_12_H_8_N_4_S)_2_(H_2_O)_2_](BF_4_)_2_. The complex shows an octa­hedral environment of the nickel cation in which the Ni^2+^ ion is located on a center of symmetry, linked to two ligands and two water molecules. In this 1:2 complex (one metal for two organic ligands) each thia­diazole ligand uses one pyridyl and one thia­diazole N atom for chelate binding. In the second pyridyl substituent, the N atom is oriented towards the same direction as the S atom of the 1,3,4-thiadiazole ring. The mean plane of the thia­diazole and pyridyl rings linked to the nickel cation forms a dihedral angle with the other pyridine ring of 18.63 (8)°. The tetra­fluorido­borate ions can be regarded as free anions in the crystal lattice. Nevertheless, they are involved in an infinite two-dimensional network parallel to (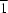
01) through O—H⋯F hydrogen bonds.

## Related literature

For Ni^II^ and Cu^II^ complexes containing a five azide ring, see: Keij *et al.* (1984 ▶). For background to similar structures, see: Bentiss *et al.* (2002 ▶, 2004 ▶, 2011 ▶); Zheng *et al.* (2006 ▶). For an improved synthesis of the ligand, see: Lebrini *et al.* (2005 ▶).
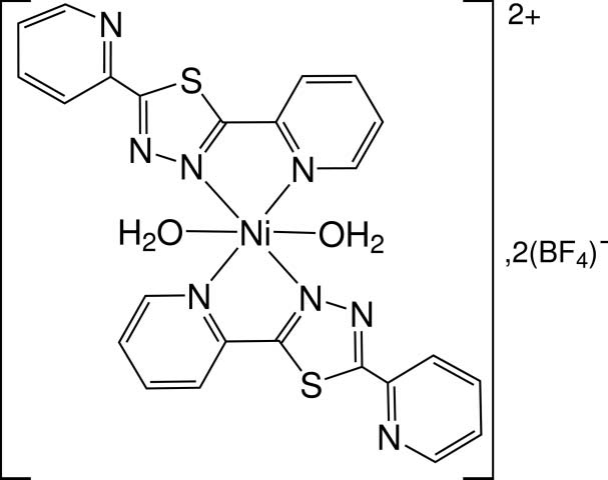


## Experimental

### 

#### Crystal data

[Ni(C_12_H_8_N_4_S)_2_(H_2_O)_2_](BF_4_)_2_
*M* *_r_* = 748.93
Monoclinic, 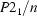

*a* = 10.8164 (15) Å
*b* = 11.0126 (13) Å
*c* = 13.2333 (16) Å
β = 101.455 (6)°
*V* = 1544.9 (3) Å^3^
*Z* = 2
Mo *K*α radiation
μ = 0.85 mm^−1^
*T* = 100 K
0.26 × 0.21 × 0.13 mm

#### Data collection

Bruker X8 APEXII CCD area-detector diffractometer
Absorption correction: multi-scan (*SADABS*; Bruker, 2005 ▶) *T*_min_ = 0.809, *T*_max_ = 0.898
28021 measured reflections
3120 independent reflections
2729 reflections with *I* > 2σ(*I*)
*R*_int_ = 0.035

#### Refinement

*R*[*F* ^2^ > 2σ(*F* ^2^)] = 0.029
*wR*(*F* ^2^) = 0.073
*S* = 1.05
3120 reflections
214 parameters
H-atom parameters constrained
Δρ_max_ = 0.60 e Å^−3^
Δρ_min_ = −0.40 e Å^−3^

### 

Data collection: *APEX2* (Bruker, 2005 ▶); cell refinement: *SAINT* (Bruker, 2005 ▶); data reduction: *SAINT*; program(s) used to solve structure: *SHELXS97* (Sheldrick, 2008 ▶); program(s) used to refine structure: *SHELXL97* (Sheldrick, 2008 ▶); molecular graphics: *ORTEPIII* (Burnett & Johnson, 1996 ▶) and *ORTEP-3 for Windows* (Farrugia, 1997 ▶); software used to prepare material for publication: *WinGX* (Farrugia, 1999 ▶).

## Supplementary Material

Crystal structure: contains datablock(s) I, global. DOI: 10.1107/S1600536811026420/dn2703sup1.cif

Structure factors: contains datablock(s) I. DOI: 10.1107/S1600536811026420/dn2703Isup2.hkl

Additional supplementary materials:  crystallographic information; 3D view; checkCIF report

## Figures and Tables

**Table 1:** Hydrogen-bond geometry (Å, °)

*D*—H⋯*A*	*D*—H	H⋯*A*	*D*⋯*A*	*D*—H⋯*A*
O1—H1*W*⋯F1^i^	0.86	1.88	2.704 (2)	160
O1—H2*W*⋯F4	0.86	1.94	2.7880 (19)	171

Symmetry code: (i) 
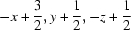
.

**Fig. 1.**
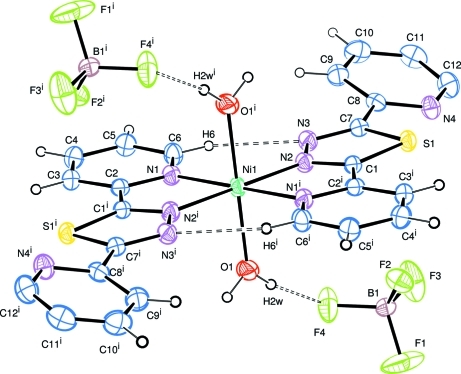
Plot of the crystal structure showing the molecules linked to the nickel cation and the counter ions, BF4-, with the atom-labelling scheme. Displacement ellipsoids are drawn at the 50% probability level. H atoms are represented as small circles. Hydrogen bonds are shown as dashed lines.

## supplementary crystallographic information

### Comment

With ligands containing a five azide ring, 3 d transition metals such as Ni^II^ and Cu^II^ have a tendency to form mono or polynuclear species (Keij *et al.*, 1984). The dinuclear species are of interest due to their potential magnetic coupling. The unpaired 3 d electrons being potentially coupled *via* the intermediary of the bridging azide ligands. The ligands related to the 1,2-diazoles with *o*-pyridine substitution at the 3 and 5 positions, such as 2,5-bis(2-pyridyl)-1,3,4 oxadiazole and thiadiazole, have been of interest for such applications. Indeed, 2,5-bis(2-pyridyl) -1,3,4-thiadiazole can be used as a molecular architect with transition metals in association with anionic Ni-ligands. In the resulting di and mononuclear complexes, a variety of coordination modes have been observed, of which the dinuclear (N`N``, N2, N``) bridging, the dinuclear (N`N``, N2, N``)2 double bridging and the monoclear (N`,*N*`)2 coordination mode are the most common and the most important ones (Scheme 1), the latter mode, the *trans* type is exclusively observed for featuring octahedral complexes.

The structures of monomeric complexes of the neutral 2,5-bis(2-pyridyl)-1, 3,4-thiadiazole derivative with divalent Zn (tetrachloride and perchlorate), Co (nitrate, perchlorate and tetrafluoborate), Ni (perchlorate), and Cu (nitrate, perchlorate) have been previously reported (Bentiss *et al.*, 2002; Bentiss *et al.*,2004; Zheng *et al.* 2006; Bentiss *et al.*, 2011). We report here the synthesis and the single-crystal structure of the new monomeric complexe formed by 2,5-bis(2-pyridyl)-1,3,4-thiadiazole with nickel tetrafluoroborate as counter ions.

The complexe shows an almost regular octahedral environment of the nickel cation in wich Ni^2+^ is located at a center of symmetry, and linked to two ligand and two water molecules as shown in Fig.1. As a matter of fact, the nickel coordination sphere is achieved by interaction with the nitrogen atom of a single pyridyl ring and with the near nitrogen of the azide group with Ni—N distances in the range of 2.052 (2)—2.132 (2) Å. Moreover, the water molecules are found in axial positions at distances and angles of Ni—O 2.120 (2) Å and all N—Ni—O angles being close to 90 °.

The dihedral angle between the thiadiazole and the pyridyl rings linked to the nickel cation is in the range of 4.16 (9)°. The mean plane of the two preceding cycles forms a dihedral angle with the (N4—C8—C9—C10—C11—C12) other pyridine ring of 18.63 (8)°. The counter ion, BF_4_^-^ is involved in an infinite two-dimensional network of O—H···F hydrogen bonds parallel to the (-1 0 1) plane (Table 1, Fig.1).

### Experimental

2,5-Bis(2-pyridyl)-1,3,4-thiadiazole ligand (noted *L*) was synthesized as described previously by Lebrini *et al.*, 2005. Ni(BF_4_)_2_6H_2_O (1.5 mmol, 0.51 g) in 8 ml of water was added to (0.42 mmol, 0.1 g) of *L* (bptd ligand) dissolved in 8 ml of ethanol. The solution was filtered and after 24 h, the colorless compound crystallized at room temperature. Crystals were washed with water and dried under vacuum. Yield: 51%. Anal. Calc. for C_24_H_20_B_2_F_8_N_8_NiO_2_S_2_: C, 38.44; H, 2.67; N, 14.95; S, 8.56; F, 20.29%. Found: C, 38.52; H, 2.75; N, 14.90; S, 8.53; F, 20.27%.

### Refinement

H atoms attached to carbon were located in a difference map but introduced in calculated position and treated as riding with C—H = 0.95 Å for the aromatic CH, with *U*_iso_(H) = 1.2 *U*_eq_ (aromatic). The O-bound H atom were initially located in a difference map and refined with O—H distance restraints of 0.86 (1). In the the last cycles of refinement, they are treated as riding on their parent O atoms with *U*_iso_(H) set to 1.2*U*_eq_(O).

### Crystal data

**Table:**

[Ni(C_12_H_8_N_4_S)_2_(H_2_O)_2_](BF_4_)_2_	*F*(000) = 756
*M**_r_* = 748.93	*D*_x_ = 1.610 Mg m^−^^3^
Monoclinic, *P*2_1_/*n*	Mo *K*α radiation, λ = 0.71073 Å
Hall symbol: -P 2yn	Cell parameters from 3120 reflections
*a* = 10.8164 (15) Å	θ = 2.7–26.3°
*b* = 11.0126 (13) Å	µ = 0.85 mm^−^^1^
*c* = 13.2333 (16) Å	*T* = 100 K
β = 101.455 (6)°	Prism, colourless
*V* = 1544.9 (3) Å^3^	0.26 × 0.21 × 0.13 mm
*Z* = 2	

### Data collection

**Table:**

Bruker X8 APEXII CCD area-detector diffractometer	3120 independent reflections
Radiation source: fine-focus sealed tube	2729 reflections with *I* > 2σ(*I*)
graphite	*R*_int_ = 0.035
φ and ω scans	θ_max_ = 26.3°, θ_min_ = 2.7°
Absorption correction: multi-scan (*SADABS*; Bruker, 2005)	*h* = −13→13
*T*_min_ = 0.809, *T*_max_ = 0.898	*k* = −13→13
28021 measured reflections	*l* = −16→16

### Refinement

**Table:**

Refinement on *F*^2^	Primary atom site location: structure-invariant direct methods
Least-squares matrix: full	Secondary atom site location: difference Fourier map
*R*[*F*^2^ > 2σ(*F*^2^)] = 0.029	Hydrogen site location: inferred from neighbouring sites
*wR*(*F*^2^) = 0.073	H-atom parameters constrained
*S* = 1.05	*w* = 1/[σ^2^(*F*_o_^2^) + (0.0302*P*)^2^ + 1.3259*P*] where *P* = (*F*_o_^2^ + 2*F*_c_^2^)/3
3120 reflections	(Δ/σ)_max_ < 0.001
214 parameters	Δρ_max_ = 0.60 e Å^−^^3^
0 restraints	Δρ_min_ = −0.40 e Å^−^^3^

### Special details

**Table:**

Geometry. All s.u.'s (except the s.u. in the dihedral angle between two l.s. planes) are estimated using the full covariance matrix. The cell s.u.'s are taken into account individually in the estimation of s.u.'s in distances, angles and torsion angles; correlations between s.u.'s in cell parameters are only used when they are defined by crystal symmetry. An approximate (isotropic) treatment of cell s.u.'s is used for estimating s.u.'s involving l.s. planes.
Refinement. Refinement of *F*^2^ against all reflections. The weighted *R*-factor *wR* and goodness of fit *S* are based on *F*^2^, conventional *R*-factors *R* are based on *F*, with *F* set to zero for negative *F*^2^. The threshold expression of *F*^2^ > 2σ(*F*^2^) is used only for calculating *R*-factors(gt) *etc*. and is not relevant to the choice of reflections for refinement. *R*-factors based on *F*^2^ are statistically about twice as large as those based on *F*, and *R*- factors based on all data will be even larger.

### Fractional atomic coordinates and isotropic or equivalent isotropic displacement parameters (Å^2^)

**Table:**

	*x*	*y*	*z*	*U*_iso_*/*U*_eq_	
B1	0.5722 (2)	0.6450 (2)	0.25602 (17)	0.0236 (4)	
F1	0.67390 (15)	0.59474 (18)	0.32316 (11)	0.0707 (6)	
F2	0.58436 (11)	0.62341 (12)	0.15219 (9)	0.0362 (3)	
F3	0.46260 (14)	0.59379 (14)	0.27433 (14)	0.0583 (4)	
F4	0.56853 (15)	0.77036 (12)	0.27479 (11)	0.0494 (4)	
C1	0.40875 (16)	0.75569 (16)	−0.02295 (14)	0.0202 (4)	
C2	0.47768 (16)	1.25248 (17)	0.06948 (13)	0.0200 (4)	
C3	0.43547 (18)	1.35965 (17)	0.10676 (14)	0.0236 (4)	
H3	0.4806	1.4333	0.1048	0.028*	
C4	0.32542 (18)	1.35711 (18)	0.14729 (15)	0.0258 (4)	
H4	0.2940	1.4292	0.1726	0.031*	
C5	0.26311 (18)	1.24770 (18)	0.14982 (15)	0.0265 (4)	
H5	0.1883	1.2433	0.1770	0.032*	
C6	0.31231 (18)	1.14360 (18)	0.11167 (15)	0.0248 (4)	
H6	0.2696	1.0686	0.1144	0.030*	
C7	0.22180 (17)	0.74859 (17)	0.04687 (14)	0.0210 (4)	
C8	0.10347 (17)	0.71642 (18)	0.08024 (14)	0.0222 (4)	
C9	0.01756 (18)	0.80507 (19)	0.09554 (16)	0.0296 (4)	
H9	0.0335	0.8887	0.0859	0.036*	
C10	−0.09264 (19)	0.7670 (2)	0.12544 (17)	0.0342 (5)	
H10	−0.1541	0.8246	0.1364	0.041*	
C11	−0.11154 (19)	0.6445 (2)	0.13902 (16)	0.0329 (5)	
H11	−0.1862	0.6166	0.1591	0.040*	
C12	−0.0195 (2)	0.5627 (2)	0.12282 (16)	0.0310 (5)	
H12	−0.0326	0.4787	0.1334	0.037*	
Ni1	0.5000	1.0000	0.0000	0.01936 (10)	
N1	0.41765 (14)	1.14452 (14)	0.07112 (12)	0.0218 (3)	
N2	0.38061 (14)	0.85992 (14)	0.01661 (12)	0.0210 (3)	
N3	0.27253 (14)	0.85652 (14)	0.05732 (12)	0.0226 (3)	
N4	0.08724 (15)	0.59655 (15)	0.09286 (13)	0.0260 (4)	
O1	0.61598 (13)	0.95553 (13)	0.14395 (11)	0.0307 (3)	
H1W	0.6844	0.9932	0.1692	0.037*	
H2W	0.6090	0.9007	0.1886	0.037*	
S1	0.30384 (4)	0.64191 (4)	−0.01275 (4)	0.02147 (12)	

### Atomic displacement parameters (Å^2^)

**Table:**

	*U*^11^	*U*^22^	*U*^33^	*U*^12^	*U*^13^	*U*^23^
B1	0.0194 (10)	0.0233 (11)	0.0268 (11)	0.0023 (8)	0.0017 (8)	0.0008 (9)
F1	0.0620 (10)	0.1029 (14)	0.0388 (8)	0.0561 (10)	−0.0106 (7)	−0.0066 (9)
F2	0.0351 (7)	0.0440 (8)	0.0285 (6)	−0.0059 (6)	0.0039 (5)	−0.0035 (6)
F3	0.0514 (9)	0.0459 (9)	0.0907 (12)	−0.0177 (7)	0.0457 (9)	−0.0236 (8)
F4	0.0759 (10)	0.0240 (7)	0.0503 (8)	−0.0093 (7)	0.0178 (7)	−0.0030 (6)
C1	0.0189 (9)	0.0170 (9)	0.0231 (9)	−0.0021 (7)	0.0004 (7)	0.0028 (7)
C2	0.0188 (9)	0.0196 (9)	0.0203 (8)	−0.0010 (7)	0.0005 (7)	0.0021 (7)
C3	0.0248 (10)	0.0184 (9)	0.0270 (10)	−0.0014 (7)	0.0034 (8)	0.0006 (8)
C4	0.0283 (10)	0.0214 (10)	0.0283 (10)	0.0025 (8)	0.0073 (8)	−0.0018 (8)
C5	0.0248 (10)	0.0258 (10)	0.0302 (10)	−0.0003 (8)	0.0090 (8)	0.0004 (8)
C6	0.0243 (9)	0.0213 (10)	0.0297 (10)	−0.0038 (8)	0.0078 (8)	0.0001 (8)
C7	0.0197 (9)	0.0197 (9)	0.0226 (9)	0.0002 (7)	0.0015 (7)	0.0009 (7)
C8	0.0194 (9)	0.0246 (10)	0.0218 (9)	−0.0032 (8)	0.0020 (7)	−0.0011 (8)
C9	0.0247 (10)	0.0270 (11)	0.0362 (11)	0.0009 (8)	0.0040 (8)	−0.0007 (9)
C10	0.0246 (10)	0.0425 (13)	0.0365 (11)	0.0058 (9)	0.0086 (9)	−0.0034 (10)
C11	0.0244 (10)	0.0476 (14)	0.0295 (10)	−0.0075 (9)	0.0116 (8)	−0.0016 (10)
C12	0.0337 (11)	0.0290 (11)	0.0331 (11)	−0.0092 (9)	0.0131 (9)	0.0009 (9)
Ni1	0.01766 (17)	0.01400 (17)	0.02659 (18)	−0.00261 (13)	0.00479 (13)	−0.00057 (13)
N1	0.0205 (8)	0.0182 (8)	0.0264 (8)	−0.0019 (6)	0.0039 (6)	0.0009 (6)
N2	0.0186 (7)	0.0179 (8)	0.0267 (8)	−0.0013 (6)	0.0048 (6)	0.0000 (6)
N3	0.0193 (8)	0.0208 (8)	0.0282 (8)	−0.0032 (6)	0.0056 (6)	0.0012 (7)
N4	0.0267 (8)	0.0233 (9)	0.0299 (8)	−0.0047 (7)	0.0099 (7)	−0.0014 (7)
O1	0.0270 (7)	0.0276 (8)	0.0337 (8)	−0.0075 (6)	−0.0030 (6)	0.0064 (6)
S1	0.0188 (2)	0.0158 (2)	0.0299 (2)	−0.00252 (17)	0.00479 (18)	0.00033 (18)

### Geometric parameters (Å, °)

**Table:**

B1—F3	1.377 (3)	C7—S1	1.7517 (19)
B1—F1	1.385 (3)	C8—N4	1.346 (3)
B1—F4	1.404 (3)	C8—C9	1.390 (3)
B1—F2	1.426 (2)	C9—C10	1.393 (3)
C1—N2	1.322 (2)	C9—H9	0.9500
C1—C2^i^	1.482 (2)	C10—C11	1.382 (3)
C1—S1	1.7137 (18)	C10—H10	0.9500
C2—N1	1.357 (2)	C11—C12	1.391 (3)
C2—C3	1.391 (3)	C11—H11	0.9500
C2—C1^i^	1.482 (2)	C12—N4	1.345 (2)
C3—C4	1.400 (3)	C12—H12	0.9500
C3—H3	0.9500	Ni1—N2	2.0515 (15)
C4—C5	1.384 (3)	Ni1—N2^i^	2.0515 (15)
C4—H4	0.9500	Ni1—O1^i^	2.1197 (14)
C5—C6	1.400 (3)	Ni1—O1	2.1197 (14)
C5—H5	0.9500	Ni1—N1^i^	2.1320 (16)
C6—N1	1.352 (2)	Ni1—N1	2.1320 (16)
C6—H6	0.9500	N2—N3	1.381 (2)
C7—N3	1.305 (2)	O1—H1W	0.8564
C7—C8	1.478 (2)	O1—H2W	0.8588
			
F3—B1—F1	108.98 (19)	C9—C10—H10	120.4
F3—B1—F4	108.44 (17)	C10—C11—C12	118.91 (18)
F1—B1—F4	109.02 (18)	C10—C11—H11	120.5
F3—B1—F2	110.41 (17)	C12—C11—H11	120.5
F1—B1—F2	109.73 (17)	N4—C12—C11	123.3 (2)
F4—B1—F2	110.22 (17)	N4—C12—H12	118.4
N2—C1—C2^i^	119.49 (16)	C11—C12—H12	118.4
N2—C1—S1	113.30 (13)	N2—Ni1—N2^i^	180.00 (9)
C2^i^—C1—S1	127.21 (14)	N2—Ni1—O1^i^	89.84 (6)
N1—C2—C3	123.10 (16)	N2^i^—Ni1—O1^i^	90.16 (6)
N1—C2—C1^i^	113.09 (16)	N2—Ni1—O1	90.16 (6)
C3—C2—C1^i^	123.81 (16)	N2^i^—Ni1—O1	89.84 (6)
C2—C3—C4	118.89 (17)	O1^i^—Ni1—O1	180.00 (8)
C2—C3—H3	120.6	N2—Ni1—N1^i^	79.22 (6)
C4—C3—H3	120.6	N2^i^—Ni1—N1^i^	100.78 (6)
C5—C4—C3	118.81 (18)	O1^i^—Ni1—N1^i^	90.02 (6)
C5—C4—H4	120.6	O1—Ni1—N1^i^	89.98 (6)
C3—C4—H4	120.6	N2—Ni1—N1	100.78 (6)
C4—C5—C6	118.84 (17)	N2^i^—Ni1—N1	79.22 (6)
C4—C5—H5	120.6	O1^i^—Ni1—N1	89.98 (6)
C6—C5—H5	120.6	O1—Ni1—N1	90.02 (6)
N1—C6—C5	123.20 (17)	N1^i^—Ni1—N1	180.0
N1—C6—H6	118.4	C6—N1—C2	117.16 (16)
C5—C6—H6	118.4	C6—N1—Ni1	128.93 (13)
N3—C7—C8	123.90 (17)	C2—N1—Ni1	113.82 (12)
N3—C7—S1	114.75 (13)	C1—N2—N3	114.22 (15)
C8—C7—S1	121.34 (14)	C1—N2—Ni1	114.28 (12)
N4—C8—C9	124.27 (17)	N3—N2—Ni1	131.42 (12)
N4—C8—C7	114.47 (16)	C7—N3—N2	110.73 (15)
C9—C8—C7	121.26 (17)	C12—N4—C8	116.68 (17)
C8—C9—C10	117.7 (2)	Ni1—O1—H1W	123.4
C8—C9—H9	121.2	Ni1—O1—H2W	131.6
C10—C9—H9	121.2	H1W—O1—H2W	105.0
C11—C10—C9	119.2 (2)	C1—S1—C7	87.00 (9)
C11—C10—H10	120.4		
			
N1—C2—C3—C4	−0.5 (3)	C3—C2—N1—Ni1	176.49 (14)
C1^i^—C2—C3—C4	178.72 (17)	C1^i^—C2—N1—Ni1	−2.83 (19)
C2—C3—C4—C5	0.7 (3)	C2^i^—C1—N2—N3	179.76 (15)
C3—C4—C5—C6	−0.1 (3)	S1—C1—N2—N3	−0.4 (2)
C4—C5—C6—N1	−0.7 (3)	C2^i^—C1—N2—Ni1	2.7 (2)
N3—C7—C8—N4	−160.31 (18)	S1—C1—N2—Ni1	−177.46 (8)
S1—C7—C8—N4	20.7 (2)	C8—C7—N3—N2	−178.38 (16)
N3—C7—C8—C9	20.3 (3)	S1—C7—N3—N2	0.6 (2)
S1—C7—C8—C9	−158.62 (15)	C1—N2—N3—C7	−0.1 (2)
N4—C8—C9—C10	−0.3 (3)	Ni1—N2—N3—C7	176.26 (13)
C7—C8—C9—C10	179.01 (18)	C11—C12—N4—C8	1.1 (3)
C8—C9—C10—C11	0.3 (3)	C9—C8—N4—C12	−0.4 (3)
C9—C10—C11—C12	0.3 (3)	C7—C8—N4—C12	−179.74 (17)
C10—C11—C12—N4	−1.0 (3)	N2—C1—S1—C7	0.63 (14)
C5—C6—N1—C2	0.9 (3)	C2^i^—C1—S1—C7	−179.58 (17)
C5—C6—N1—Ni1	−175.27 (14)	N3—C7—S1—C1	−0.72 (15)
C3—C2—N1—C6	−0.2 (3)	C8—C7—S1—C1	178.31 (16)
C1^i^—C2—N1—C6	−179.56 (16)		

Symmetry codes: (i) −*x*+1, −*y*+2, −*z*.

### Hydrogen-bond geometry (Å, °)

**Table:**

*D*—H···*A*	*D*—H	H···*A*	*D*···*A*	*D*—H···*A*
O1—H1W···F1^ii^	0.86	1.88	2.704 (2)	160.
O1—H2W···F4	0.86	1.94	2.7880 (19)	171.

Symmetry codes: (ii) −*x*+3/2, *y*+1/2, −*z*+1/2.
